# Molecular organization of recombinant human-Arabidopsis chromosomes in hybrid cell lines

**DOI:** 10.1038/s41598-021-86130-4

**Published:** 2021-03-30

**Authors:** Yikun Liu, Yeng Mun Liaw, Chee How Teo, Petr Cápal, Naoki Wada, Kiichi Fukui, Jaroslav Doležel, Nobuko Ohmido

**Affiliations:** 1 Graduate School of Human Development and Environment, Kobe University, Kobe, Hyogo 657-8501 Japan; 2 Centre for Research in Biotechnology for Agriculture, Universiti Malaya, 50603 Lembah Pantai, Kuala Lumpur, Malaysia; 3 Institute of Experimental Botany of the Czech Academy of Sciences, Centre of the Region Haná for Biotechnological and Agricultural Research, Šlechtitelů 31, 779 00 Olomouc, Czech Republic; 4 Graduate School of Technology, Industrial and Social Sciences, Tokushima University, Tokushima, Tokushima 770-8503 Japan; 5 Graduate School of Pharmaceutical Sciences, Osaka University, Suita, Osaka 565-0871 Japan

**Keywords:** Cell biology, Genetics, Molecular biology

## Abstract

Although plants and animals are evolutionarily distant, the structure and function of their chromosomes are largely conserved. This allowed the establishment of a human-Arabidopsis hybrid cell line in which a neo-chromosome was formed by insertion of segments of Arabidopsis chromosomes into human chromosome 15. We used this unique system to investigate how the introgressed part of a plant genome was maintained in human genetic background. The analysis of the neo-chromosome in 60- and 300-day-old cell cultures by next-generation sequencing and molecular cytogenetics suggested its origin by fusion of DNA fragments of different sizes from Arabidopsis chromosomes 2, 3, 4, and 5, which were randomly intermingled rather than joined end-to-end. The neo-chromosome harbored Arabidopsis centromeric repeats and terminal human telomeres. Arabidopsis centromere wasn’t found to be functional. Most of the introgressed Arabidopsis DNA was eliminated during the culture, and the Arabidopsis genome in 300-day-old culture showed significant variation in copy number as compared with the copy number variation in the 60-day-old culture. Amplified Arabidopsis centromere DNA and satellite repeats were localized at particular loci and some fragments were inserted into various positions of human chromosome. Neo-chromosome reorganization and behavior in somatic cell hybrids between the plant and animal kingdoms are discussed.

## Introduction

The unique structure of eukaryotic chromosomes played important roles in genome evolution, speciation, and transmission of genetic information to progenies. The study of chromosome functions, including replication, segregation, gene expression, and inheritance, is necessary to reveal the differences between plant and animal chromosome organization and function. Understanding chromosome conservation among different organisms can contribute not only to genetics, but also to studies of the biosynthesis and genome evolution of the organisms.

Cell fusion is a useful technique commonly used in biomedical research for gene mapping^1, 2^. It has also been used to produce hybridoma cells through fusion of murine myeloma cells with human lymphocytes to produce monoclonal antibodies^3^. However, cell fusion often induces chromosomal instability. Production of somatic hybrid cell lines is often accompanied by chromosome loss whose mechanism is not clear. Human-rodent somatic cell hybrids are useful tools for mapping human chromosomes and understanding gene functions. Human-mouse hybrid cell lines^4^ showed preferential loss of human chromosomes and at least 75% of the human component was lost. The clones had a relatively stable karyotype, and to some extent, the human chromosome content was found to be dependent on the culture conditions. After the hybrid population was formed, Weiss and Green^4^ noted that continued subculture resulted in slow elimination of the human chromosome component. However, no evidence of chromosomal rearrangement was found in a majority of hybrid cell lines.

In a mouse chimeric line, several human chromosome fragments were retained and expressed in chimeric mice^5^. A human chromosome fragment of about 1–2 Mb carrying human Ig genes was maintained as an independent chromosome in the chimeric mice, and chromosomal rearrangement occurred in some of the clones. Most of the chimeras retained the transferred chromosome fragment in all tissues and one of the derived fragments showed germline transmission up to the F4 generation with no observable phenotype defects.

The stability of different human chromosomal fragments in different cell types has also been studied. Shinohara et al.^6^ constructed a mouse library carrying human chromosome fragments. The estimated sizes were 5–20 Mb for human chromosome fragments hCF-2 and hCF-11 and 90 Mb for hCF-14. Interchromosomal rearrangements were not detected in mouse embryonic stem cells and all the transferred human chromosomes were maintained as independent extra copies. hCF-14 had high stability, but hCF-2 and hCF-11 were relatively unstable in mouse embryonic stem cells. The unstable hCF-2 and more stable hCF-14 were tested in human fibrosarcoma HT1080, HeLa, and chicken DT40 B lymphoma cells under prolonged culture. The retention rate of hCF-14 was relatively high in HT1080, HeLa, and DT40, whereas hCF- 2 was lost with different kinetics in HT1080 and HeLa. The stability of hCF-14 in various cell types may indicate the role of specific DNA elements in determining the retention stability.

Genome behaviour in hybrids has been investigated among evolutionarily very distant organisms^7^. Allshire et al.^8^ produced a yeast-mouse hybrid cell line carrying a large yeast chromosomal segment in a mouse background. The spheroplast fusion cell line was originally made by fusing fission yeast *Schizosaccharomyces pombe*, carrying an integrated mammalian selectable marker, with a mouse tumor cell line^8^. This cell line carried an insertion of *S. pombe* DNA at a single location on mouse chromosome 10^9^. McManus et al.^9^ investigated the composition of the yeast transgenomes and the modification of chromatin structure of the yeast DNA in the mouse cells. Chromatin of a large foreign DNA introduced into mammalian cells showed distinct condensation from that of the surrounding host mammalian DNA. The insertions that originated from foreign DNA were coincident with the presence of high levels of the heterochromatin marker histone H3 trimethylated on lysine 9^10^. In some cases, such as protoplast fusion of interspecific yeast hybrids, chimeric chromosomes may arise because of chromosome replacement, rearrangement, and recombination in chimeric hybrid yeasts^11^.

Previous interkingdom cell fusion lines of human HeLa cells with carrot protoplasts^12^ and hen erythrocytes with yeast protoplasts^13^ were maintained as heterokaryons that existed as multinucleate cells, although some interphase nuclei were fused in the human-carrot hybrid cells. An interphylum somatic hybrid of human HeLa cells with mosquito *Aedes aegypti* by ultraviolet-inactivated *Sendai* virus were maintained as mononucleate hybrid cells in ten subcultures with the loss of insect chromosomes, but a near diploid number of human chromosomes^14^.

In our previous work, we created a human-plant hybrid cell line by fusing *A. thaliana* protoplasts and human HT1080 cells^15^. The human-Arabidopsis hybrid cells stably maintained the plant-derived neo-chromosomes (PD chromosomes) and number of Arabidopsis genes were expressed in the human genetic background^15^. The results revealed that Arabidopsis-derived sequences fused to human chromosome 15 via a process called terminal translocation. However, the detailed characterization of its genome has not been performed yet.

In this study, we investigated the structure of the Arabidopsis-derived neo-chromosome as well as changes in its structure after 240 days culture. Recently, next generation high-throughput sequencing (NGS) and copy number variation (CNV) analysis have been applied to investigate how alien chromosomes maintain and function in host organism. Structural variation detection methods, including fluorescence in situ hybridization (FISH), microarrays, and WGS, have been used to investigate chromosomal changes^16^. We also performed whole-genome sequencing on 60- and 300-day-old cell cultures; that is, 60 and 300 days after the initial cell-protoplast fusion event. The reorganization of integrated Arabidopsis genome regions in the hybrid cell line was investigated by FISH and WGS coupled with CNV and DNA repeat analysis. The results provided new insights into the neo-chromosome formation, and the maintenance and alteration of plant DNA in human genetic background.

## Results

### Elimination and reorganization of the Arabidopsis genome in a human-plant hybrid cell line

**Figure 1:**
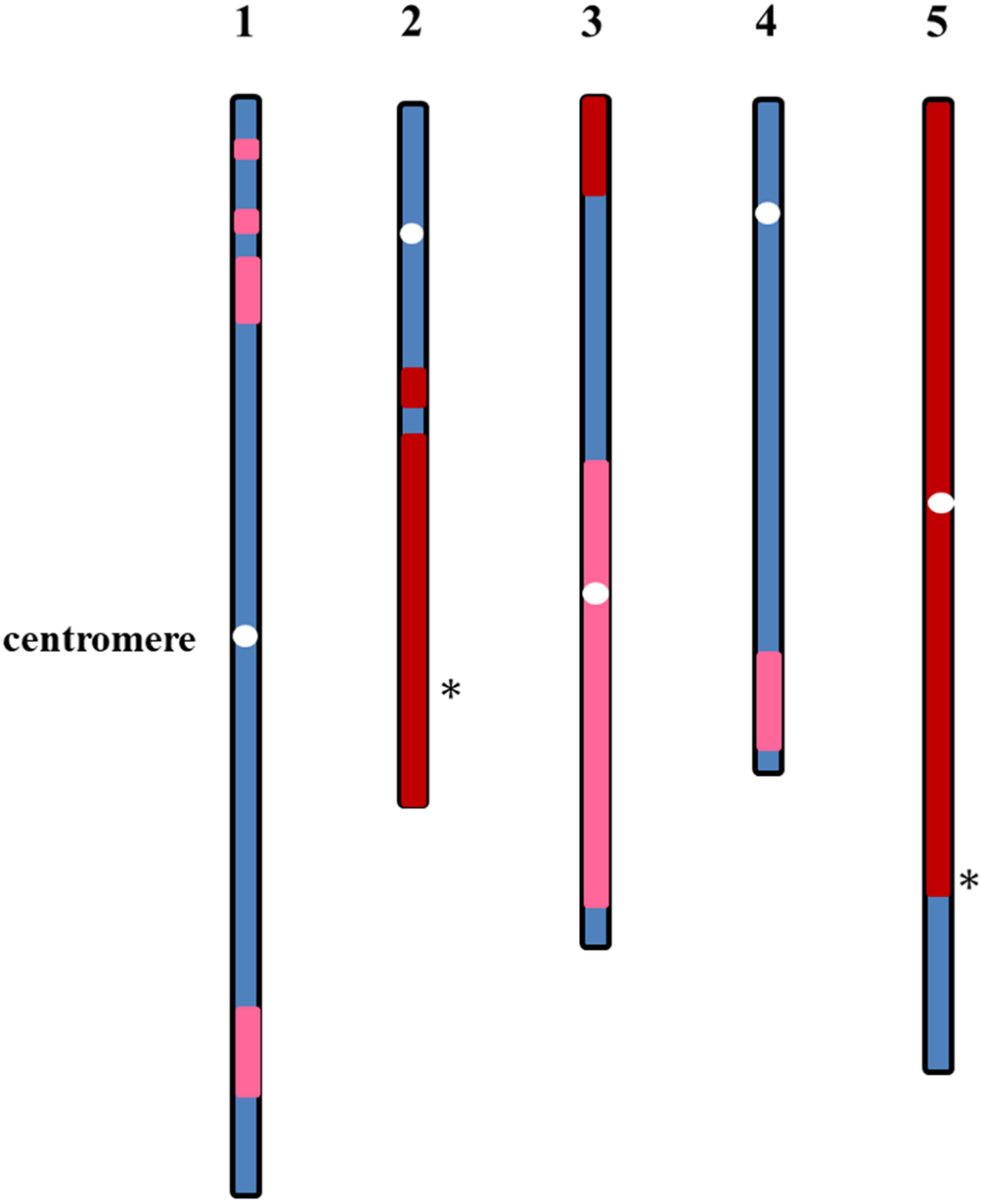
*Arabidopsis thaliana* sequences present in the 60-day and 300-day-old hybrid cell line. The idiogram shows the regions larger than 200 kb of the Arabidopsis chromosomes that were 60-day present (pink + red), 300-day-old present (red) and absent (blue) in the hybrid cell line. White ovals indicate centromere positions; asterisks (*) indicate fusion positions that join Chr2 and Chr5 in the hybrid genome.

To characterize DNA sequences in the hybrid cell line, and molecular organization of the human-Arabidopsis neo-chromosome in particular, whole-genome Illumina sequencing was done on 60- and 300-day-old hybrid cell lines at 50 × and 30 × coverage, respectively. Mapping DNA sequence reads of 60-day hybrid cell to the *A. thaliana* genome assembly (TAIR10) revealed the presence of fragments from all Arabidopsis chromosomes, including fragments of the short and long arms (Chr1), complete centromeric repeat regions (Chr3 and Chr5) and telomeric regions (Chr2, Chr3, and Chr5) (pink and red zones in Fig. 1). However, 300-day hybrid cell showed considerable elimination of Arabidopsis sequences from Chr1, Chr3 and Chr4 (pink zones in Fig. 1), while remaining alien fragments of Chr2 and Chr5 were unaffected by the elimination process (red zones in Fig. 1). Majority of long arm of Chr2 present in 60-day cell remained entirely in 300-day-old culture, together with more than 80% of Chr5 including its centromeric region was kept in 300-day-old culture (Table 1). As DNA sequences of the entire long arm of Chr3 was absent in the de novo assembly WGS data of the 300-day hybrid cell line, we concluded that the retained centromeric repeats originated from Chr5. When compared with the microarray data from the original hybrid cell line^15^, the WGS of the Arabidopsis genome performed on our 60-day cell line correlated well with the previous microarray results. Their microarray analysis identified more than 400 gene loci (Wada et al.^15^, Fig. S5) in five Arabidopsis chromosomes and all of these loci were also detected in our WGS analysis, as represented in Fig. 1.

**Table 1:** *Arabidopsis thaliana* genome regions present in the 60-and 300-day-old hybrid cell lines.

*A. thaliana* chromosome	Location in *A. thaliana* genome (bp)	Sequence length (bp)	60-day	300-day	Markers
1	1,341,549–1,850,658	509,109	+	−	
1	3,339,470–3,876,463	536,993	+	−	
1	4,933,240–6,138,109	1,204,869	+	−	
1	16,509,811–17,148,282	638,471	+	−	
1	25,384,354–27,494,134	2,109,780	+	−	
2	7,507,287–8,318,501	811,214	+	+	
2	9,554,225–19,698,289	10,144,064	+	+	BAC T1J8^a^
3	96–2,693,711	2,693,615	+	+	BAC T9J14^a^, *Bsd*^b^, GFP^c^
3	8,348,362–22,733,522	14,385,160	+	−	
4	9,257,087–9,867,323	610,236	+	−	
5	0–21,629,605	21,629,605	+	+	BAC T22P22^a^

Markers of Arabidopsis genomic region: ^a^Bacterial artificial chromosome (BAC); ^b^Blasticidin S resistance (*Bsd*-resistance) gene and ^c^Green fluorescence protein (GFP) gene localized by Wada et al.^15^ in hybrid cell. + : present, −: not present.

To identify fusion breakpoints in the neo-chromosome, structural variations were called using DELLY^17^ and the predicted breakpoints were confirmed by PCR followed by Sanger sequencing. The results showed that a segment of Arabidopsis Chr5 at position 21,627,782 joined a segment of Chr2 at position 15,076,777 of their original TAIR10 coordinates (marked by asterisk in Fig. 1, Supplementary Fig. S1 and Table S1). The results were consistent for both the 60- and 300-day-old cell lines. The interrogated junction comprised terminal 198 bp from Chr5 fragment and 64 bp from the middle of the Chr2 fragment joined in a 3′ to 5′ orientation (Supplementary Fig. S1), while remaining parts of Chr2 were scattered on different positions of neo-chromosome and other human chromosome. The structural variations detected using DELLY were supported by four split-reads and seven discordant paired-end reads that aligned to both chromosomes.

Calculated using SAMtools, the WGS data showed a higher proportion of the Arabidopsis genome in the 60-day-old culture (55 Mbp, 46%) than in the 300-day-old culture (29 Mbp, 24%), confirming that Arabidopsis-derived genome fragments in the hybrid cell line were lost during the 240 days of culture. To further investigate this phenomenon, we analyzed CNV and characterized DNA repeat content in the Arabidopsis-derived fragments. To characterize Arabidopsis DNA repeat composition, we analyzed de novo assembled genome of the hybrid cell line to identify Arabidopsis repeats using RepeatMasker^18^. In general, all known Arabidopsis repeat families (e.g., retroelements, DNA transposons) were present in the hybrid genome in lower numbers compared to the wild-type Arabidopsis genome (Supplementary Fig. S2 and Supplementary Table S2). The repeat composition was similar in the 60- and 300-day-old hybrid cell lines, suggesting that the elimination of repeat families occurred at an earlier stages of cell culture. Interestingly, the cumulative length of satellite repeats was measurably higher in both the 60- and 300-day-old cell lines (4,879,652 bp and 3,740,017 bp, respectively) compared to the Arabidopsis (976,448 bp) and human (1,485,509 bp) genomes.

**Figure 2:**
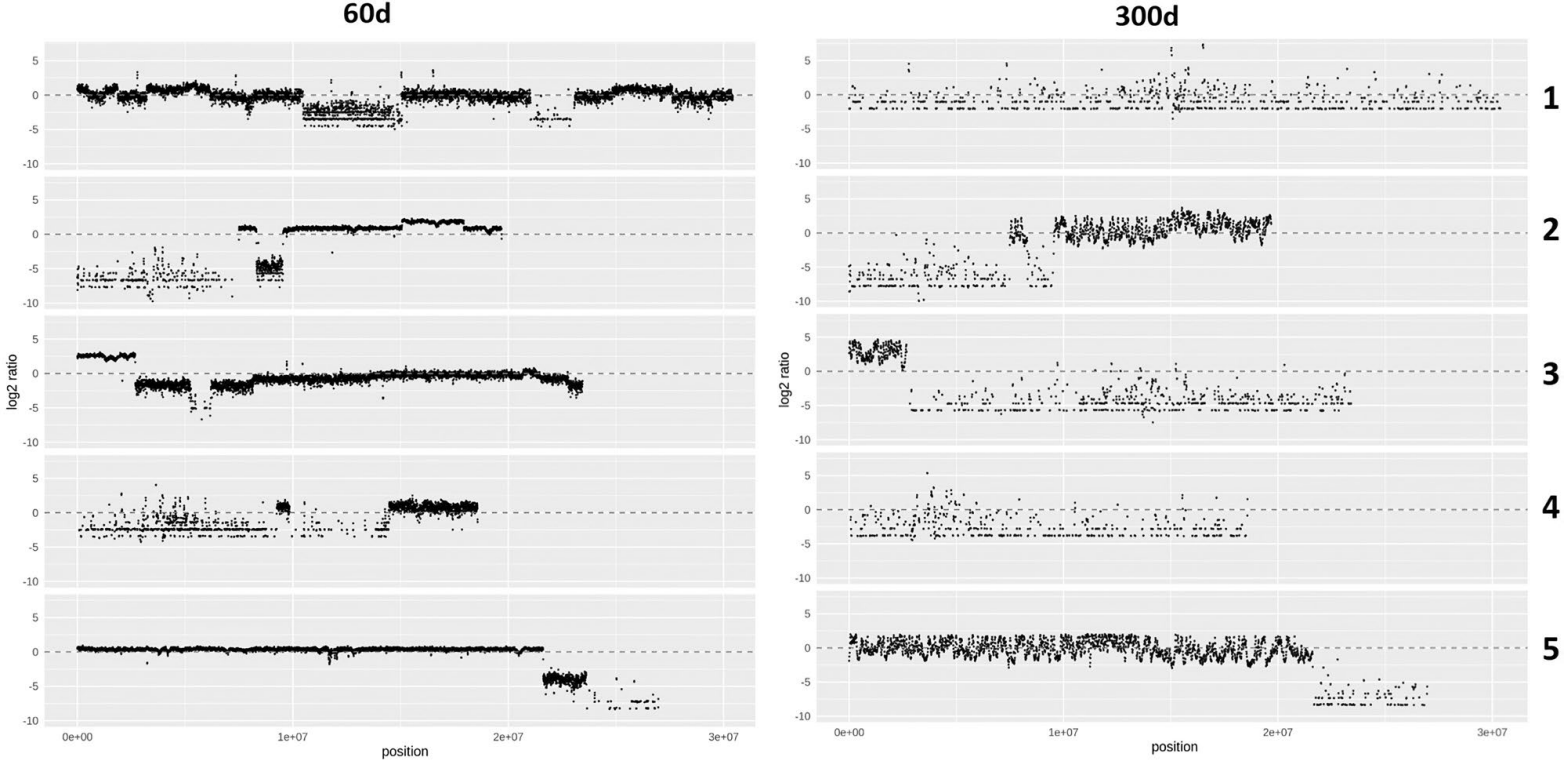
Copy number variation of Arabidopsis genome fragments in the 60- and 300-day-old hybrid cell lines. Log2 ratio of zero (gray dashed line) indicates diploid level. X-axis: DNA sequence of Arabidopsis pseudomolecules; Y-axis: log2 ratio relative to the Arabidopsis reference genome TAIR10. Numbers on the right side of the graphs indicate Arabidopsis chromosome numbers. Differences in plot density were caused by the adjustment of window size in CNV-seq according to coverage depth.

Changes in copy number of the introgressed Arabidopsis genome segments were determined by CNV-seq^19^ after normalization with the Arabidopsis reference genome TAIR10 (Fig. 2). Plot density was adjusted by CNV-seq according to the coverage depth. The WGS data from the 60-day-old hybrid cell line showed that the genome of hybrid cell line contained DNA fragments from all Arabidopsis chromosomes (Fig. 1, Table 1). CNV-seq data showed a relatively stable copy number state for 60-day-old hybrid cell, with no more than two copy number states detected for Arabidopsis Chr2 and Chr5. The CNV cluster with the log2 value of -4 around the end of the long arm of chromosome 5 indicates that the copy number difference between this cluster in wild type Arabidopsis and hybrid cells is at least 16 fold different. This suggested that the majority of the hybrid cells in the highly heterogeneous hybrid cell population were lacking in this chromosome region.

Conversely, CNV was higher in the 300-day-old hybrid cell line and large fluctuations in copy number state were detected in DNA fragments from all Arabidopsis chromosomes. Considerably less DNA from Chr1, Chr4 and Chr3 was found in the 300-day-old cell line. Larger variation in DNA copy number, especially for Chr5-derived sequences, indicated DNA loss and amplification. The log2 ratio of Chr3 in the 60-day-old cell line indicated 2- to eightfold variation in copy number, whereas in the 300-day-old cell line, the copy number varied up to 32-fold. Contrary to the segmented manner that indicated block-by-block fluctuations of copy number observed in 60-day-old culture, in the 300-day-old culture continuous copy number fluctuations with an unclear segment boundary were detected. In entire human chromosome, 300-days old hybrid cell showed a slightly higher fluctuation compared to the 60-days old cell (Supplementary Fig. S3). However, in the 300-days old hybrid cell line, the copy number changes of the human chromosome (log2 ratio; 1 to -2.5) are smaller in the comparison to the Arabidopsis chromosome 3 (0 to 5) and 5 (2.5 to -2.5), respectively. No copy number gains on the human chromosome 15 and others during the 240-days culture was observed. Therefore, it is deduced that genome instability occurred in the whole hybrid genome in cell culture.

### Neo-chromosome structure and cytogenetic landmarks

Neo-chromosomes were classified into three subtypes, PD type-T, type-S and type-A based on the number and orientation of the Arabidopsis genomic signals on the neo-chromosomes^15^. We used cell culture followed by FISH analysis with Arabidopsis centromeric probes to estimate cell heterogeneity by identifying the neo-chromosomes, classified as PD type-T, type-A, and/or type-S. This was performed on both 60-day and 300-day cell cultures in this study. 95% type-T and only 5% type-A chromosomes in 21 chromosome spreads (Supplementary Fig. S4) were detected in the 300 days cell cultures. No type-S was detected. To ensure reliability of the neo-chromosomes detection, we performed the same FISH analysis on a 60-day cell line only two days after cells were revived from cold storage. We observed a similar trend, with 88% of type-T and 12% of type-A chromosomes in a total of 25 chromosome spreads counted (Supplementary Fig. S4). No type-S was observed.

**Figure 3:**
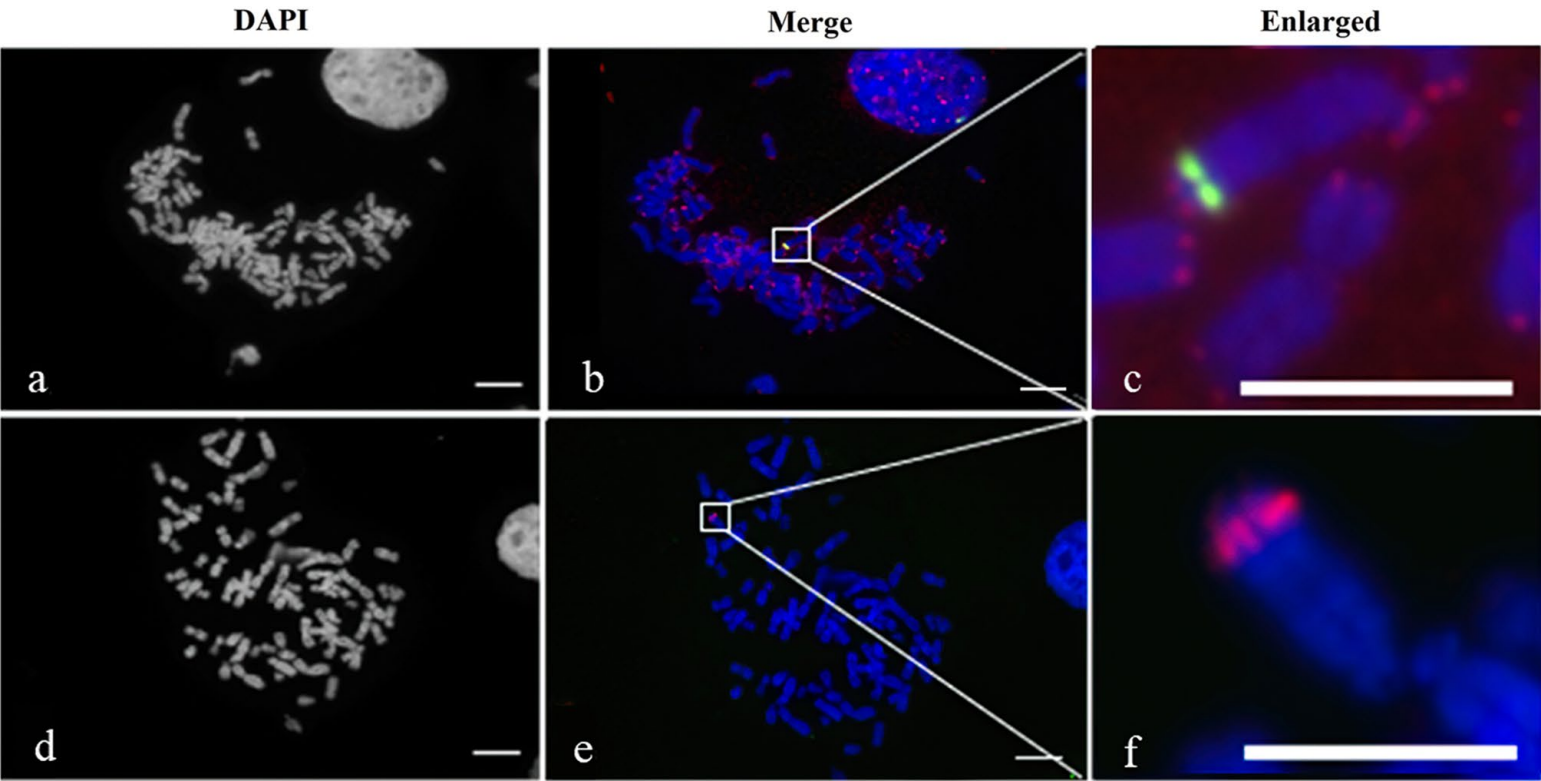
Genomic distribution of DNA repeats in the 300-day hybrid cell line. Fluorescence in situ hybridization (FISH) was done on mitotic metaphase plates using probes for Arabidopsis centromere (Atcen, green) and human telomere (red) (**a–c**), and the Atcen (red) and Arabidopsis telomere (green) (**d–f**). Chromosomes were counterstained using DAPI (gray pseudocolor in **a**,**d** and blue in **b**,**c**,**e**,**f**). Scale bars, 10 µm (**a**,**b**,**d**,**e**) and 5 µm (**c**,**f**).

We applied FISH on mitotic metaphase spreads to detect the orientation of the Arabidopsis chromosome fragments, confirm telomere type and centromere origin of the neo-chromosome in the 300-day-old hybrid cell line (Fig. 3). FISH failed to detect Arabidopsis telomeric repeats (TTTAGGG)n^20^ in cells of the 300-day-old culture (Fig. 3d–f), which indicated the Arabidopsis telomere sequence units were absent, or only few were present and below a detection limit of FISH. On the other hand, the human telomere probe (TTAGGG)n was detected at both termini of the neo-chromosome with clear and strong hybridization signals (Fig. 3a–c). Moreover, a weak human telomere signal was detected in the proximal region of the neo-chromosome in the 60 day-old cell line, but this signal was not detected in the 300-day old cell line, indicating a rapid loss of this locus during the culture. A strong hybridization signal of Arabidopsis centromere (Atcen, 180 bp unit) repeats was observed next to the human telomere loci. The human telomere loci on the neo-chromosomes appeared near the Atcen signals probably because of low spatial resolution due to high chromosome condensation. These Atcen repeat signals were much stronger than in the wild-type Arabidopsis mitotic metaphase plates, suggesting amplification of the Atcen sequence in the hybrid cell line (Fig. 3c,f).

**Figure 4:**
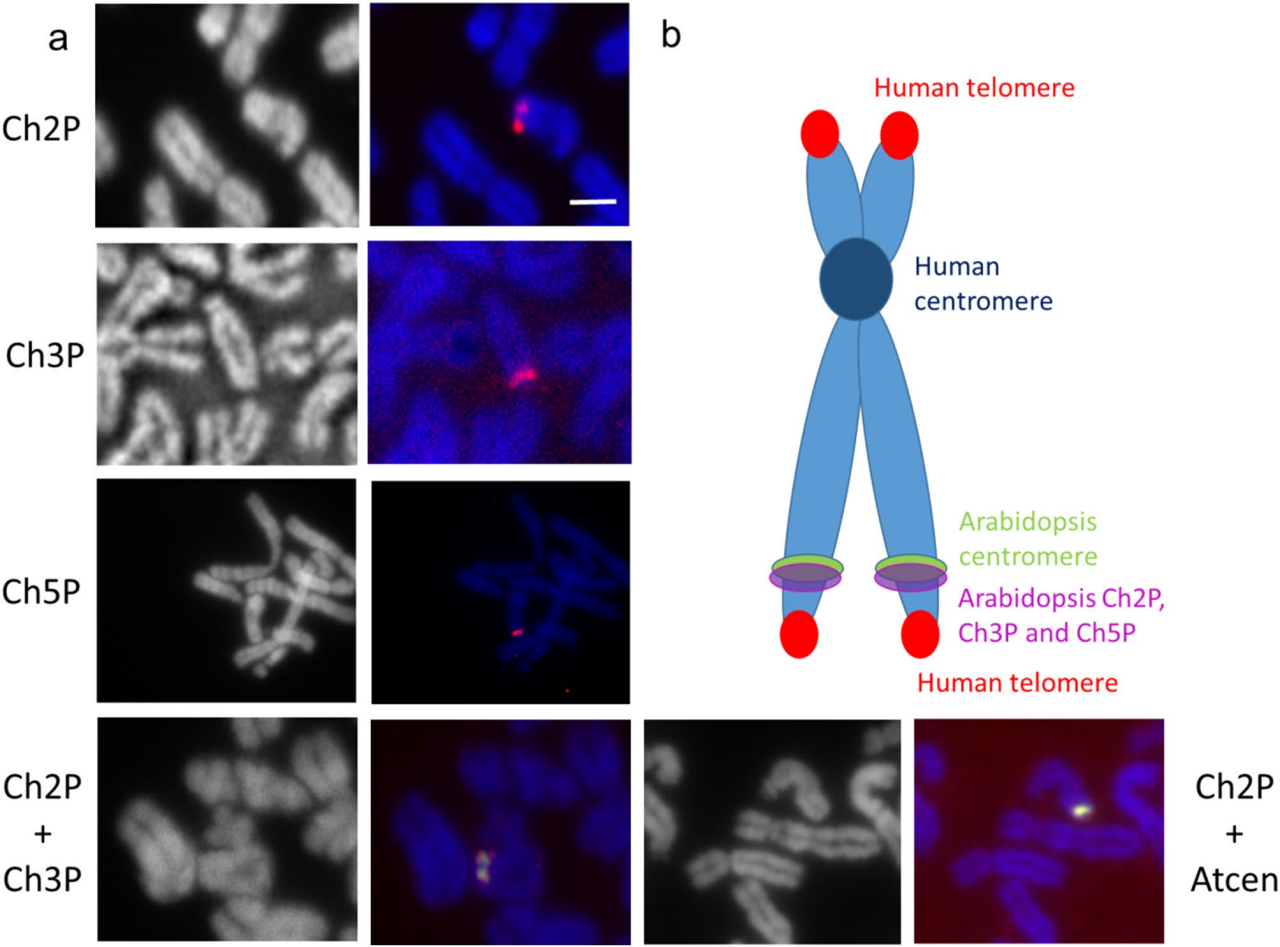
Molecular organization of the neo-chromosome. (**a**) Localization of Arabidopsis genome segments in mitotic metaphase chromosomes by FISH using chromosome-specific pooled probes singly, Ch2P (red), Ch3P (red), Ch5P (red), the combination Ch2P (red)/Ch3P (green), and Ch2P (red)/AtCen (green). Chromosomes were counterstained using DAPI (gray pseudocolor or blue). (**b**) Diagrammatic representation of the neo-chromosome. Scale bar, 5 µm.

To localize particular Arabidopsis DNA sequences on the neo-chromosome, pooled probes Ch2P, Ch3P, and Ch5P were used for FISH. Each pooled probe consisted of five PCR amplicons targeting single-locus DNAs covering approximately 5 kb physical distance in the TAIR10 genome (Supplementary Table S3). Their hybridization signals were located close to each other on the distal part of neo-chromosome. To determine the orientation of Ch2P and Ch3P, they were labelled with a different fluorochrome and detected simultaneously by FISH on the metaphase chromosome of 300-day-old hybrid cell line (Fig. 4a). The Ch2P fluorescence signal overlapped with the Ch3P and Arabidopsis centromere signals, respectively (Fig. 4a, bottom plates). The hybridization signals of each single probe were detected on the same regions (Supplementary Fig. S5). It would be due to the short physical distances between the Arabidopsis genome segments as represented by FISH analysis using the pooled probes.

The FISH results are summarized in Fig. 4b. The neo-chromosome was formed from human chromosome 15 (HSA15)^15^ into which Arabidopsis genome segments had integrated. The mitotic neo-chromosome resembled the normal HSA15 structure, with human telomere signals on both termini and a human centromere in the primary constriction. A compact region included the Atcen sequence localized on the long arm of a distal part of HSA15 and Arabidopsis genome sequences from Chr2, Chr3, and Chr5 with human-specific telomeres.

### Multiple localization of the Ch2-2 probe suggests translocation and amplification of Arabidopsis genome segments in the human genome

**Figure 5:**
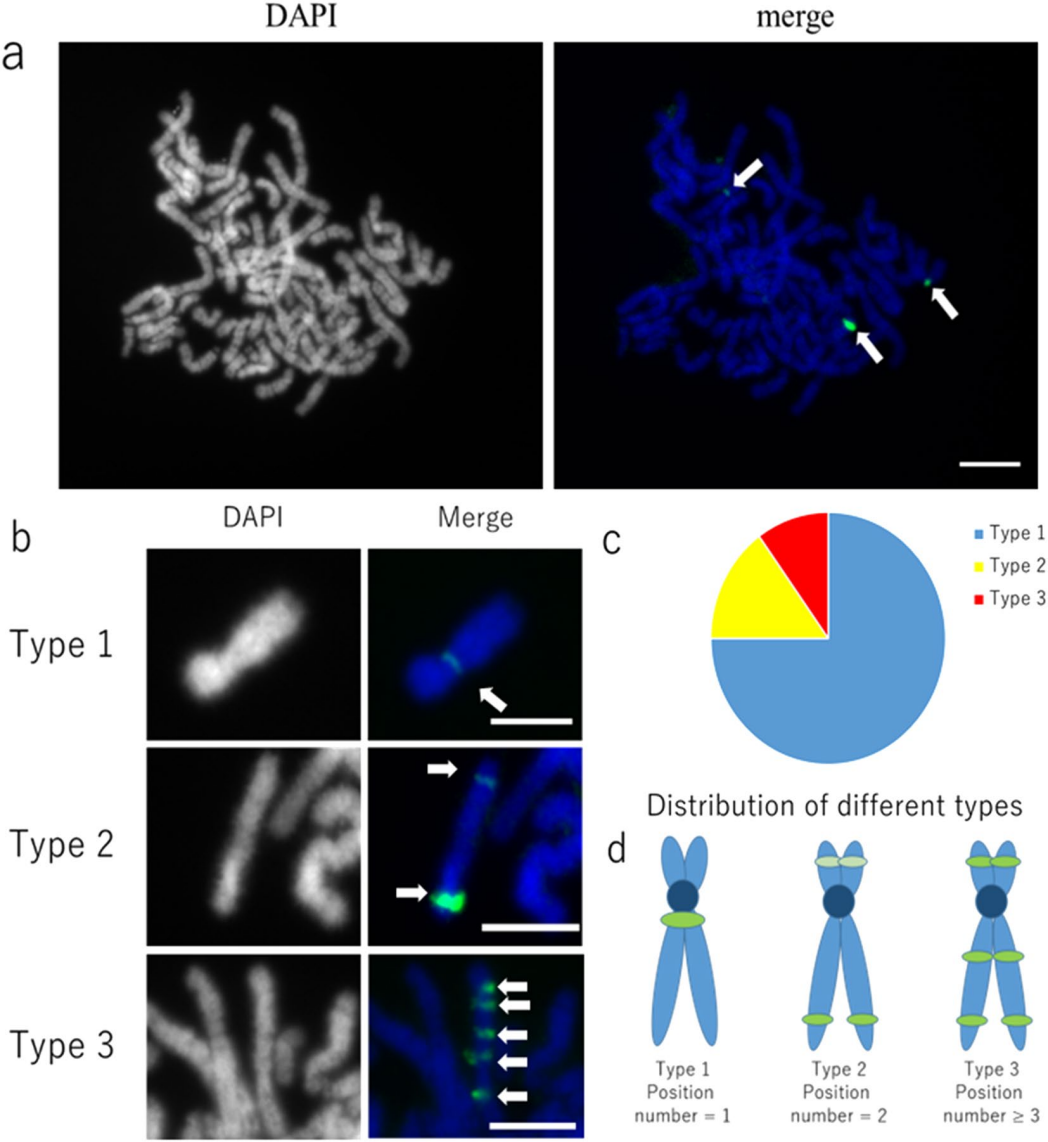
Localization of Arabidopsi*s* genome segment Ch2-2 by FISH to human chromosomes in the hybrid cell line. (**a**) Probe Ch2-2 (green) hybridized to different chromosomes (arrows). Chromosomes were counterstained using DAPI (gray pseudocolor or blue). (**b**) Different types of chromosomal location of the Ch2-2 probe. (**c**) Relative proportion of chromosomes with different Ch2-2 signal distributions. 20 cells were examined by FISH for Ch2-2 localization. (**d**) Diagrammatic representation of the three types of chromosomes. Scale bars, 5 µm.

To check the distribution of each of the Arabidopsis single-copy genome segments, three 400–500 bp long DNA probes (Ch2-2, Ch3-2, and Ch5-2) were selected from 15 single copy probes for FISH analysis (Supplementary Table S3). The Ch3-2 and Ch5-2 probes always localized only on the expected locus on the neo-chromosome in both the 60- and 300-day-old cell lines (Supplementary Fig. S5). In the 60-day-old hybrid cell line, only one expected signal for Ch2-2 was observed and no probe hybridization signal was found in the original hybrid cell line HT1080 (Supplementary Fig. S6). Surprisingly, in the 300-day-old cell line, the Ch2-2 hybridization signal was observed on three to ten positions within the genome, not only on the neo-chromosome, but also on other human chromosomes (Fig. 5a). Moreover, the Ch2-2 signal positions on the human chromosomes varied; some were close to the centromere while others were near telomeric regions (Fig. 5b). The chromosomes of hybrid cell line were thus classified into three types according to the number of Ch2-2 hybridization signals and the frequency of each type (Fig. 5c,d). Type 1 (75% of the chromosomes) had only one signal next to the centromere, type 2 (15% of the chromosomes) had two signals near telomeres, and type 3 (10% of the chromosomes) had more than two signals. The signals found on the human chromosomes other than those on HSA15 suggested that translocation and duplication occurred during the course of cell culture. The variable count of hybridization signals in different cells indicated chromosome instability in the hybrid cell line.

**Figure 6:**
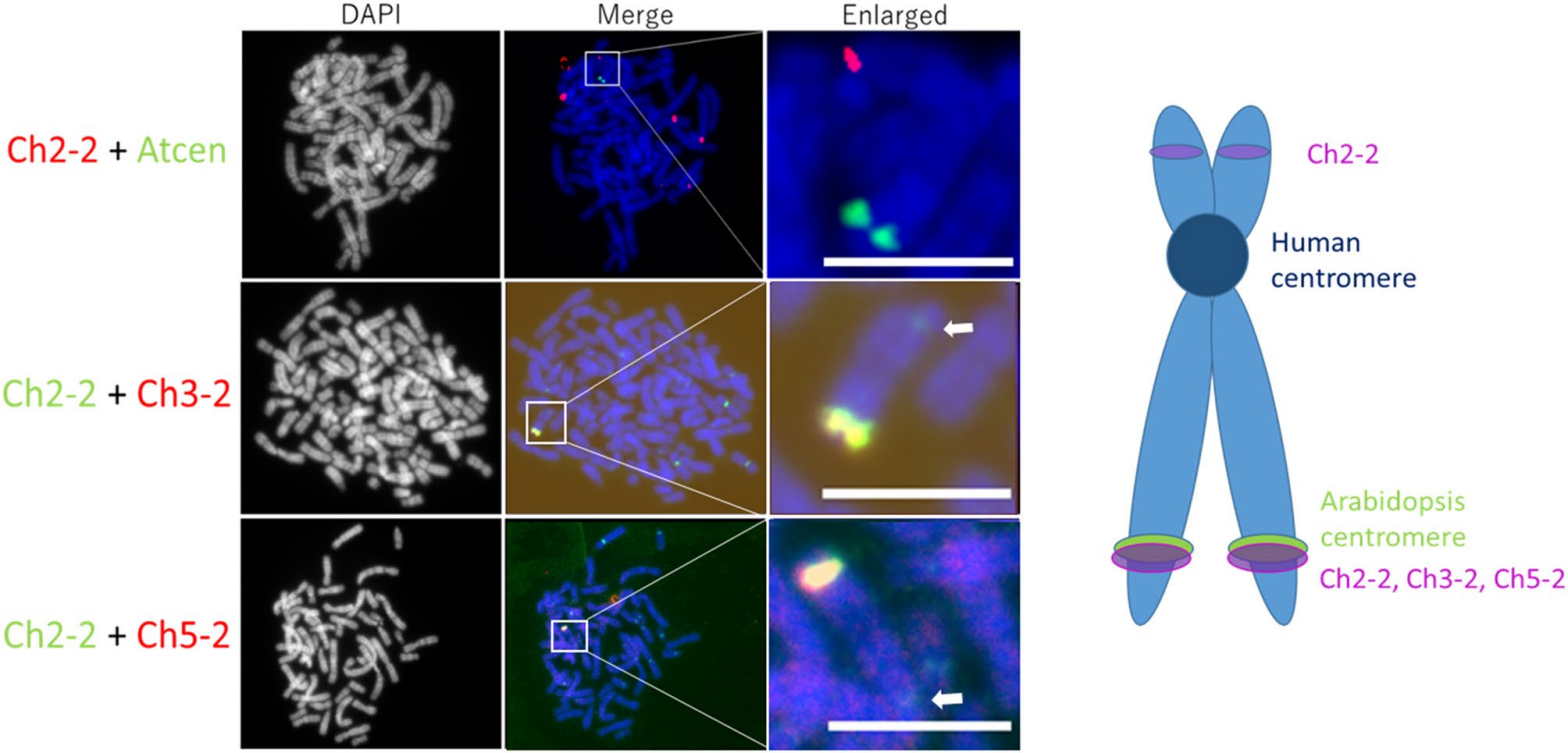
Simultaneous localization of Arabidopsis probes Ch2-2, Ch3-2, Ch5-2 and Arabidopsis centromere (Atcen) probe by FISH on metaphase chromosomes in the hybrid cell line. Arrows indicate weak Ch2-2 signals on the short chromosome arm. Scale bars, 5 µm.

Simultaneous FISH with Ch2-2 and Atcen probe resulted in multiple hybridization signals of Ch2-2, but none of these signals detected on the neo-chromosome with Atcen signal (Fig. 6). Conversely, when pooled probes for Arabidopsis Ch2P were combined with Atcen probes, the hybridization signals overlapped and Ch2P probes cross hybridized in the same locus with the Atcen probes (Fig. 4, bottom right). Although Ch3-2 and Ch5-2 were supposed to be close to Ch2-2, the Ch3-2 and Ch5-2 probes did not block the Ch2-2 signal (middle and bottom plates in Fig. 6), possibly because the Ch2-2 locus was closer to the Atcen region or even overlapped on metaphase chromosomes.

## Discussion

We report new insights into the long-range molecular organization of a unique neo-chromosome that originated in a human-plant hybrid cell line. The neo-chromosome was first described by Wada et al*.*^15^ and comprises human chromosome 15 and regions of Arabidopsis chromosomes 2, 3, and 5 as originally revealed by BAC-FISH and microarray analysis. We confirmed this principal composition of the neo-chromosome and we have identified Arabidopsis genome sequence maintained in the hybrid cells using illumina sequencing (Fig. 1, Table 1). We have also employed FISH to determine the orientation of the inserted Arabidopsis chromosome segments and to outline the evolution of the neo-chromosome during the culture of the hybrid cell line (Figs. 3, 4, 5).

Our work revealed complex chromosomal rearrangement which occurred during the formation and maintenance of the neo-chromosome in the human-Arabidopsis hybrid cell line. The Arabidopsis-derived neo-chromosome was assembled by the fusion of Arabidopsis chromosome fragments to human chromosome 15, and contained human and Arabidopsis centromeres, two human telomeres, and highly rearranged chromosomal fragments of Arabidopsis chromosomes 2, 3, and 5 after 300 days of culture. A comparison of the 60- and 300-day-old cell lines revealed that fragments of the Arabidopsis genome were lost and their number decreased from 46% (55 Mb) in the 60-day-old cell line to 24% (29 Mb) in the 300-day-old cell line and that the remaining fragments underwent large fluctuations in copy numbers (Fig. 2). WGS, CNV and FISH revealed that the alien Arabidopsis genome was unstable as culture time progressed and some of the plant DNA fragments were lost. However, some Arabidopsis chromosome fragments, like the T-type, survived long term culture but the repeat sequences present in the Arabidopsis genome became larger than usual.

The WGS results revealed a fragmented manner in which Arabidopsis genome was retained (Supplementary Fig. S1) as well as non-conventional fusion breakpoint structure joining the terminal part of Arabidopsis chromosome 5 and interstitial part of chromosome 2 (Supplementary Fig. S1). The observed overlap of the introgressed Arabidopsis sequences (Figs. 4, 6) indicated that complex processes were involved in the insertion of the Arabidopsis genome fragments. It is also possible that duplication of genome fragments occurred in the hybrid cell line. However, the mechanism responsible for the rearrangement of the Arabidopsis regions in the hybrid cells could not be determined by sequencing because of the low coverage (average 1.7 ×) of the relatively small Arabidopsis regions in the human genome background. For a mouse-yeast hybrid, Fitz-James et al.^10^ confirmed that the inserted yeast DNA was composed of rearranged DNA with 10–100 kb fragments (median length 20 kb) of contiguous yeast genome in the mouse fusion cells.

A comparison of the 60- and 300-day-old cell lines revealed that at least 50% of the Arabidopsis genome regions were eliminated during culture (Fig. 2) and a majority of these sequences were from Chr1, Chr3, and Chr4. Moreover, the copy number variation of the remaining Chr2, Chr3, and Chr5 sequences in the 300-day-old cell line was larger than in 60-day-old (Fig. 2). In particular, the region of Chr3 that contained the selective marker genes, blasticidin S (*Bsd*) resistance and green fluorescence protein (GFP) was amplified dramatically up to 32-fold and the Chr5 fragment that contained the centromere repeats was also amplified. These results indicate that the function of these two regions may be selectively controlled. Because cell survival in the media including blasticidin S requires the presence of the *Bsd-*resistance gene that is located on Chr3 in this hybrid cell line^15^, all cells presumably contained the Chr3 fragment that contained the resistance gene. However, the reason for preferable retention of other Arabidopsis chromosomal regions is not clear. Previous studies of a human-rodent somatic cell hybrid described preferential loss of complete mouse or human chromosomes^4, 21^. Somatic cell hybrids often exhibit random chromosome loss in cultures, but the mechanism is yet to be elucidated^22^. Fission yeast *S. pombe* chromosomes were integrated into mouse cells by somatic fusion^9^, and the yeast DNA was found to be integrated into a single site within a mouse chromosome and maintained there. The alien yeast DNA replicated as an independent unit using their own replication system in late S phase, and the replication timing was not synchronized with that of the flanking mouse DNA.

Wada et al.^15^ conducted a cytological analysis of the human-Arabidopsis 60-day-old hybrid cell line and concluded that the neo-chromosome originated via a process of end-to-end fusion of HSA15 with Arabidopsis chromosomes, performing three types of plant-derived chromosome (type T, S and A) during the culture process. In the 300-day old culture, the majority of the neo-chromosomes were type-T, most probably due to its preferential retention, as the plant chromosomal region was attached to HSA 15, allowing it is to pass on during cell replication. The previous report by Wada et al.^15^ relied on a continuous culture of the hybrid cell line immediately after the fusion event. The cells used in our experiments derived from the original cells but after 4.5 years after storage at − 80 °C. This long-term cold storage may explain the discrepancies observed in the 60-day cell line between previous Wada et al.^15^ and this study. We think that the freeze–thaw process might have caused the retention of the more stable type-T neo-chromosome, rather than type-S and type-A. Previous studies also demonstrated that cryopreservation of sperm could induce cell injury with increased DNA fragmentation^23, 24^. Based on the morphology of the translocated chromosome, we concluded that the acrocentric chromosome observed could only have been human chromosome 13, 14 or 15. Considering the previous results of Wada et al.^15^, we therefore estimated that the neo-chromosome was human chromosome 15 in both 60- and 300-day cell line.

Clusters of human telomeric sequence (TTAGGG)n were observed at both termini of the neo-chromosome and also in its interstitial region. The human telomeric sequence on the distal end of the HSA15 short arm could be the original human telomere. However, in the 300-day-old hybrid cell line, the Arabidopsis telomeric repeat sequence (TTTAGGG)n and human telomeric repeat sequence (TTAGGG)n were not detected in the proximal parts of the neo-chromosome (Fig. 4). Following tandem chromosome fusions, interstitial telomeric sequences have often been found in several plants and animal species^25–28^ and were not functional^25^. The observed shortening or loss of the tandem repeats may be caused by polymerase replication slippage or unequal crossing over^29, 30^.

Another mechanism leading to genomic rearrangements is the formation of a dicentric chromosome containing two functional centromeres^31^, and our neo-chromosome consisted of two centromeres, one from human and one from Arabidopsis. Maciejowski et al.^32^ showed that dicentric chromosomes that caused the formation of chromatin bridges in mitosis can lead to chromothripsis (*i.e.*, chromosome shattering) through cycles of breakage and rejoining called breakage-fusion-bridge cycles. Gisselsson et al.^33^ proved that breakage-fusion-bridge events can lead to genetic heterogeneity in tumors. This possibility was also noted by Wada et al.^15^ because human histone H3 variant centromere protein (CENP)-A was detected on two subtypes of the neo-chromosome (type S and A) by immunostaining. The expression of Arabidopsis centromere-specific histone H3 (CENH3) was not detected in the microarray data. In 300-day-old hybrid cell, CENH3 gene on Chr1 was completely eliminated. The amplified Arabidopsis centromere derived from Chr5 wasn’t functional as Arabidopsis centromere in the 300-day-old hybrid cell line. Dicentric chromosomes are usually formed as a product of telomere fusion. When the two centromeres of a dicentric chromosome migrate to opposite mitotic spindle poles during, breakage-fusion-bridge cycle is initiated, resulting in complex chromosome rearrangements^34^. However, because the neo-chromosome contained only one active human centromere, the complex chromosomal rearrangement was most probably not a consequence of dicentric chromosome formation.

Interestingly, several inter-chromosomal translocations were found by FISH using Arabidopsis chromosome-specific DNA probes (Table S3) and karyotype heterogeneity was observed in the hybrid cell population (Fig. 5). One possible reason behind these translocations is the involvement of transposable elements, which can be activated when a host genome fails to suppress their activity^35^. As the sequence of Ch2-2 fragment is single-locus, it did not coincide with any transposable element in *A. thaliana* Repeat Maps^36^. Interspecific translocation also was reported in mouse-human cell lines, where the long arm of human chromosome 17, or a portion of human X chromosome was translocated to a mouse chromosome^1, 2^. Friend et al.^37^ also described several mouse-human hybrids that showed interspecific translocation. They observed the presence of the same hybrid chromosome in majority of the cells within a clone, although they didn’t investigate the maintenance of the hybrid chromosome under long culture times^1, 37^.

Considering the arguments mentioned above and the complex molecular composition of the neo-centromere, a highly probable mechanism of its origin seems to be chromothripsis, where up to hundreds of structural rearrangements are acquired in a short time^38^. This process is characterized by extensive genomic rearrangements and oscillating patterns of DNA copy number levels^39^. Our WGS data revealed that Arabidopsis DNA was retained in fragments (Fig. 2) and FISH data showed highly overlapped regions of Arabidopsis Chr2, Chr3, and Chr5 (Figs. 4, 6). Thus, we speculate that chromothripsis occurred during cell fusion when the Arabidopsis genome was shattered, stitched, and randomly inserted into the human genome.

In this study, oscillations of copy number states occurred in all the Arabidopsis chromosomes in the hybrid cell lines, and occurred randomly across all chromosomes. A large difference in copy number variation with rapid elimination of large genome regions from the 60- to 300-day-old cultures indicated genomic instability in the hybrid line. The criterion for the occurrence of chromothripsis is clustered chromosomal rearrangements confined in localized genomic regions^40^. However, Gu et al*.*^41^ reported that chromothripsis-like rearrangement also occurred beyond confined regions involving two or three chromosomes. The cause for the occurrence of chromothripsis is still unclear, with possible reasons including dicentric centromere, centromere inactivation or a genomic stress^42^. It is probable that a genomic shock due to the fusion of divergent genomes may lead to large-scale genome restructuring. Fragmented retention of Arabidopsis genome sequences, highly overlapped Arabidopsis chromosomal regions on the neo-chromosome, and multiple insertion of Arabidopsis chromosomal fragments into human genome all may contribute to stress.

To conclude, we demonstrated that fragments of plant genomes can be maintained in human cells. However, a majority of plant DNA sequences are eliminated after long culture because of genomic stress generated from the cross-kingdom hybridization. We suggest that the Arabidopsis genome was fragmented by chromothripsis^43^ and its fragments were integrated into human chromosomes. Determination of gene expression and epigenetic modifications of the Arabidopsis genome fragments will be important to test how cross-kingdom conservation of mechanism regulate genome function.

## Materials and methods

### Plant material and DNA extraction

Seeds of *Arabidopsis thaliana* cv. Columbia (2*n* = 10) from Kobe University were sterilized in 1 mL 10% (v/v) kitchen bleaching solution (KAO, Tokyo, Japan) for 30 min, rinsed several times, vernalized at 4 °C for 2–3 days and germinated on 0.5 × Murashige and Skoog (MS) medium (Wako, Tokyo, Japan) supplemented with 0.5% agar for 10–15 days at 25 °C. DNA was extracted from seedlings in DNA Suisui buffer (RIZO Inc., Tsukuba, Japan) and ethanol precipitated.

### Cell culture

The human-Arabidopsis hybrid cell line that contained a neo-chromosome (PD chromosome) was obtained by Wada et al.^15^ from 60- and 149-day-old cultures. 60-days and 149-days cell cultures originated from cell stocks kept in storage frozen at − 80 °C for 5.5 and 4.5 years, respectively. The 60-day cell culture was used for experiments almost immediately, after only a few days of stabilization. The 300 day culture originated from cells previously grown for 149-day and stored frozen. These cells were revived and propagated every 2–3 days in culture for an additional 150 days before performing WGS and FISH analysis. The hybrid cell line was cultured in Dulbecco's Modified Eagle Medium (DMEM) (Gibco, Carlsbad, California, USA) supplemented with 10% fetal bovine serum (FBS) (Gibco) and 6 μg/mL blasticidin S (Bsd) (KNF, Tokyo, Japan) at 37 °C in a 5% CO_2_ incubator.

### DNA extraction from hybrid cells

Genomic DNA was extracted from 60- and 300-day-old hybrid cells according to Miller et al.^44^. Briefly, the cells were lysed, protein digested, RNase treated, and ethanol precipitated. Approximately 8 million cells at 90% confluency in 10-cm plates were lysed with 300 µL nuclei lysis buffer (10 mM Tris-HCl, 400 mM NaCl, 2 mM EDTA, pH 8.2) and digested at 37 °C overnight with 20 µL 10% SDS and 50 µL proteinase K solution (1 mg protease K in 1% SDS and 2 mM EDTA). Then, 100 µL 5 M NaCl was added and shaken vigorously for 15 s to precipitate the protein. The sample was pelleted at 13,000 × *g* for 15 min and the supernatant was transferred to a new tube. Next, 10 µL RNase A (10 mg/mL) was added to 200 µL of the solution and incubated at 37 °C for 1 h. Two volumes of absolute ethanol were added and the tube was inverted several times and kept overnight at − 80 °C. Then, the sample was spun at 13,000 × *g* for 30 min at 4 °C. The supernatant was discarded and the pellet was washed with 70% cold ethanol and centrifuged again at 13,000 × *g* for 5 min at 4 °C. The supernatant was discarded and the pellet was left to air dry before being resuspended in distilled water. DNA concentration was measured with Qubit 4 Fluorometer (Thermo Fisher Scientific, Waltham, MA, USA) and its integrity was checked by gel electrophoresis.

### Whole-genome sequencing

Genomic DNA isolated from the 60-day-old hybrid cell line was sequenced on Illumina NovaSeq 6000 instrument with 150-bp paired-end reads at the Institute of Experimental Botany^45^. The sequencing library was prepared using NEBNext Ultra II DNA Library Prep Kit for Illumina (E7645) (New England BioLabs Inc., Ipswich, USA) with dual index primers and four rounds of PCR amplification. DNA fragments of 400–1000 bp were selected using Pippin Prep (Sage Science Inc., Beverly, MA, USA). Genomic DNA isolated from the 300-day-old hybrid cell line was sequenced by Macrogen Corp. (Kyoto, Japan) on Illumina NovaSeq 6000 instrument with 150-bp paired-end reads. The sequencing library was prepared using TruSeq DNA PCR-Free kit (Illumina, Inc., Tokyo, Japan).

### NGS data processing

Quality control was performed using FastQC^46, 47^ and adapter trimming and quality filtering were performed with Fastp and/or Trimmomatic^46, 48^. To avoid unspecific mapping, the filtered reads were mapped to combined genomes of human (GRCh38) and *A. thaliana* (TAIR10) with BWA-MEM v0.7.17^18^. Duplicated reads were removed with Picard (http://broadinstitute.github.io/picard) after mapping. Reads that aligned to the *A. thaliana* genome were extracted for downstream processing. The proportion of Arabidopsis genome in the hybrid cells was calculated using SAMtools^49^. Long contiguous Arabidopsis region present in hybrid cell was determined using BEDTools^50^ coupled with visual inspection of sequencing reads with reference to CNV data. Figure 1 and Table 1 were generated using BEDtools merge by first merging book-ended/ flanking regions and overlapping features. For each of the merged regions, neighbouring regions within 500 bp were merged again, and only regions longer than 10 kb were retained. From these regions, neighbouring regions within 500 kb were again merged and regions exceeding 200 kb were retained. Finally, only regions larger than 500 kb were shown in Fig. 1 and Table 1, respectively.

### Detection of fusion junction and structural variation

To detect fusion junctions between the introgressed Arabidopsis chromosome segments, structural variations were called using DELLY^17, 46^ and filtered with supporting split reads or paired-end reads of at least three pairs. Translocated chromosomal regions were extracted to identify junction sequences between Arabidopsis chromosome fragments. Primers flanking the junction region were designed using Primer3.

### De novo assembly

Sequence reads were assembled using Minia 3^51^ with optimal k-mer determined using KmerGenie (1.7051)^52^. The reads from the 60- and 300-day-old hybrid cell lines were assembled with k83-mer and k73-mer, respectively. The assembled genomes were quality checked using QUAST (5.0.2). The assembled genomes from the 60- and 300-day-old hybrid cell lines consisted of 986,822 contigs with N50 of 3,922 bp and 935,237 contigs with N50 of 4,281 bp, respectively.

### Characterization of DNA repeats

De-novo assembled genomes were masked with RepeatMasker (v1.332)^18^ to identify Arabidopsis DNA repeat families (Supplementary Table S2). Simple repeats and low complexity repeats were not considered because of considerable overlap with the human genome. The Arabidopsis and human reference genomes were masked with the same Arabidopsis repeat library for comparison.

### Copy number variation

CNV of Arabidopsis genome regions in the hybrid cell line was determined by CNV-seq^19^ using default parameters with a minimum sliding window of 4, p-value 0.001, and log2 threshold 0.6. The bin size is pre-determined by CNV-seq based on the read depth. The window size was 9054 bp and 19,748 bp for the 60- and 300-day-old cultures, respectively. The log2 ratio was obtained by normalizing with the Arabidopsis reference genome TAIR10 using data accessed from run DRR188057 of Bioproject PRJDB8649 in the Sequence Read Archive (SRA) database (https://www.ncbi.nlm.nih.gov/sra/). Publicly available WGS data of HT1080 (SRR9931532) was obtained from the SRA database and used as the reference for both 60-day and 300-day hybrid cells to compare copy number variation changes in the hybrid genome. The analysis output from CNV-seq was then plotted in a programing language ‘R’ (Fig. 2).

### Mitotic cell arrest

Hybrid cells (5 × 10^7^ per 10-cm dish) were seeded and subjected to 2.5 mM thymidine treatment after 1 day of culture. After 24 h, the medium was exchanged with DMEM supplemented with 10% FBS and 6 μg/mL Bsd. The cells were cultured for 6 h and then recultured for 24 h in the presence of 2.5 mM thymidine. Following this, the medium was exchanged with DMEM supplemented with 10% FBS and 6 μg/mL Bsd, and synchronized cells were cultured for 7 h, after which the synchronized cells were arrested with 0.05 µg/mL colcemid (NACALAI TESQUE, Kyoto, Japan) for 4 h^53^.

### Chromosome preparation for FISH

Synchronized cells were spun down for 10 min at 1000 g, re-suspended in 1 mL Hypotonic Buffer N (10 mM Hepes pH 7.5, 2 mM MgCl_2_, 25 mM KCl) containing 1 mM DTT and 1 mM PMSF (added just before use), and incubated on ice for 1 h. The suspension was then centrifuged for 10 min at 500 g. Pelleted cells were fixed in 1 mL fresh Carnoy’s solution (3:1 ethanol:glacial acetic acid). Then, 10 µL of chromosome suspension in Carnoy’s solution was dropped onto a clean glass slide that was 25 cm below the pipet tip. Before drying out, a drop (10–15 µL) of fresh Carnoy’s solution was applied to the slide to spread the chromosomes.

### Preparation of probes for FISH

Fifteen probes for Arabidopsis single-copy genome regions, Arabidopsis centromere repeats (Atcen, 180 bp), and telomeric short repeats of human (TTAGGG) and Arabidopsis (TTTAGGG) were amplified by PCR to generate FISH probes^54, 55^. The primer sequences used for the PCRs are listed in Supplementary Table S4. Purified PCR products were labelled with Cy3-dUTP (GE Healthcare, Chicago, Illinois, USA) and Fluorescein-12dUTP (Roche Diagnostics GmbH, Mannheim, Germany) using a Nick Translation kit (Roche Diagnostics). The average fragment size of the probes was 100–300 bp. The Arabidopsis-specific probes are listed in Supplementary Table S4. Three compound probes also were prepared: Ch2P (contains Ch2-1–Ch2-5), Ch3P (Ch3-1–Ch3-5) and Ch5P (Ch5-1–Ch5-5) (Supplementary Table S3).

### Fluorescence in situ hybridization (FISH)

Slides were pre-treated in 1% formaldehyde/PBS at room temperature for 5 min followed by washing 2 times in PBS for 5 min each and rinsing briefly in water. Then, the slides were denatured in 70% formamide in 2 × SSC at 70 °C for 4 min followed by dehydration in 70% then 100% cooled ethanol for 5 min each and air-dried. Hybridization mixtures containing labelled probes and HB buffer (50% formamide/2 × SSC/10% dextran sulfate MW 500,000) were denatured at 80 °C for 10 min and immediately cooled on ice. Next, 10 μL of the hybridization mix was applied onto each slide, covered with a glass coverslip (18 × 18 mm), and sealed. The slides were incubated at 37 °C for 1–2 days in a humid dark box. After hybridization, the coverslips were removed, and the slides were washed 3 times in 20% formamide in 2 × SSC at 42 °C for 5 min each, followed by washing three times in 0.1 × SSC at 42 °C for 5 min each. Then, the slides were washed three times in 2 × SSC at room temperature for 5 min each, briefly rinsed in distilled water, and air-dried in the dark. After drying, the slides were sealed in 10–15 μL Vectashield (Vector Lab, Inc., CA, USA) containing 1.5 μg/mL DAPI and covered with 24 × 24 mm glass coverslips. FISH images were photographed directly using a SPOT RT3 CCD camera (SPOT imaging solutions, Inc., MI. USA) with a BX60 microscope (Olympus, Tokyo, Japan) using standard Olympus optical filter sets. Image analyses were done using ImageJ 1.52a (NIH).

## Supplementary Information

Supplementary Information

## Data Availability

All data generated or analyzed during this study are included in this article and supplemental data. The human-Arabidopsis cell line used in the study was used under license from the corresponding author of Ref. 15, and cannot be shared by the authors. Readers wishing to use this cell line for research should direct their requests to the authors of Ref. 15.

## References

[1] Elsevier SM (1974). Assignment of the gene for galactokinase to human chromosome 17 and its regional localisation to band q21–22. Nature.

[2] Boyd Y (1987). Characterization and use of somatic cell hybrids with interspecific translocations involving the human X chromosome. Ann. Hum. Genet..

[3] Samoilovich SR, Dugan CB, Macario AJ (1987). Hybridoma technology: new developments of practical interest. J. Immunol. Methods.

[4] Weiss MC, Green H (1967). Human-mouse hybrid cell lines containing partial complements of human chromosomes and functioning human genes. Proc. Natl. Acad. Sci. U. S. A..

[5] Tomizuka K (1997). Functional expression and germline transmission of a human chromosome fragment in chimaeric mice. Nat. Genet..

[6] Shinohara T (2000). Stability of transferred human chromosome fragments in cultured cells and in mice. Chromosome Res..

[7] Wang DY, Kumar S, Hedges SB (1999). Divergence time estimates for the early history of animal phyla and the origin of plants, animals and fungi. Proc. Biol. Sci..

[8] Allshire RC (1987). A fission yeast chromosome can replicate autonomously in mouse cells. Cell.

[9] McManus J (1994). Unusual chromosome structure of fission yeast DNA in mouse cells. J. Cell Sci..

[10] Maximilian HFJ (2020). Large domains of heterochromatin direct the formation of short mitotic chromosome loops. eLife.

[11] Kishida M, Muguruma T, Sakanaka K, Katsuragi T, Sakai T (1996). Chromosomal deletion or rearrangement in chimeric hybrids of Saccharomycopsis fibuligera and Saccharomyces diastaticus obtained by cell fusion. J. Ferment. Bioeng..

[12] Dudits D, Rasko I, Hadlaczky G, Lima-de-Faria A (1976). Fusion of human cells with carrot protoplasts induced by polyethylene glycol. Hereditas.

[13] Ahkong QF (1975). Fusion of hen erythrocytes with yeast protoplasts induced by polyethylene glycol. Nature.

[14] Zepp HD, Conover JH, Hirschhorn K, Hodes HL (1971). Human-mosquito somatic cell hybrids induced by ultraviolet-inactivated Sendai virus. Nat. New Biol..

[15] Wada N (2017). Maintenance and function of a plant chromosome in human cells. ACS Synth. Biol..

[16] Weckselblatt B, Rudd MK (2015). Human structural variation: mechanisms of chromosome rearrangements. Trends Genet..

[17] Rausch T (2012). DELLY: structural variant discovery by integrated paired-end and split-read analysis. Bioinformatics.

[18] Smit, A. F. A., R., H. & P., G. *RepeatMasker Open-4.0.*, http://www.repeatmasker.org/ (2013–2015).

[19] Xie C, Tammi MT (2009). CNV-seq, a new method to detect copy number variation using high-throughput sequencing. BMC Bioinform..

[20] Richards EJ, Ausubel FM (1988). Isolation of a higher eukaryotic telomere from Arabidopsis thaliana. Cell.

[21] Croce CM (1976). Loss of mouse chromosomes in somatic cell hybrids between HT-1080 human fibrosarcoma cells and mouse peritioneal macrophages. Proc. Natl. Acad. Sci. U. S. A..

[22] Kucherlapati RS, Ruddle FH (1975). Mammalian somatic hybrids and human gene mapping. Ann. Intern. Med..

[23] Johnston SD, Zee YP, López-Fernández C, Gosálvez J (2012). The effect of chilled storage and cryopreservation on the sperm DNA fragmentation dynamics of a captive population of koalas. J. Androl..

[24] Kopeika J, Thornhill A, Khalaf Y (2015). The effect of cryopreservation on the genome of gametes and embryos: principles of cryobiology and critical appraisal of the evidence. Hum. Reprod. Update.

[25] Meyne J (1990). Distribution of non-telomeric sites of the (TTAGGG)n telomeric sequence in vertebrate chromosomes. Chromosoma.

[26] Reimann N (1994). Evidence that metacentric and submetacentric chromosomes in canine tumors can result from telomeric fusions. Cytogenet. Cell Genet..

[27] Lee C, Sasi R, Lin CC (1993). Interstitial localization of telomeric DNA sequences in the Indian muntjac chromosomes: further evidence for tandem chromosome fusions in the karyotypic evolution of the Asian muntjacs. Cytogenet. Cell Genet..

[28] Schubert I, Schriever-Schwemmer G, Werner T, Adler ID (1992). Telomeric signals in robertsonian fusion and fission chromosomes: implications for the origin of pseudoaneuploidy. Cytogenet. Cell Genet..

[29] Schlötterer C, Tautz D (1992). Slippage synthesis of simple sequence DNA. Nucleic Acids Res..

[30] Messier W, Li SH, Stewart CB (1996). The birth of microsatellites. Nature.

[31] Gascoigne KE, Cheeseman IM (2013). Induced dicentric chromosome formation promotes genomic rearrangements and tumorigenesis. Chromosome Res..

[32] Maciejowski J, Li Y, Bosco N, Campbell PJ, de Lange T (2015). Chromothripsis and kataegis induced by telomere crisis. Cell.

[33] Gisselsson D (2000). Chromosomal breakage-fusion-bridge events cause genetic intratumor heterogeneity. Proc. Natl. Acad. Sci. U. S. A..

[34] MacKinnon RN, Duivenvoorden HM, Campbell LJ (2011). Unbalanced translocations of 20q in AML and MDS often involve interstitial rather than terminal deletions of 20q. Cancer Genet..

[35] Aravin AA, Hannon GJ, Brennecke J (2007). The Piwi-piRNA pathway provides an adaptive defense in the transposon arms race. Science.

[36] Kapitonov VV, Jurka J (1999). Molecular paleontology of transposable elements from Arabidopsis thaliana. Genetica.

[37] Friend KK, Dorman BP, Kucherlapati RS, Ruddle FH (1976). Detection of interspecific translocations in mouse-human hybrids by alkaline Giemsa staining. Exp. Cell Res..

[38] Stephens PJ (2011). Massive genomic rearrangement acquired in a single catastrophic event during cancer development. Cell.

[39] Zhang CZ (2015). Chromothripsis from DNA damage in micronuclei. Nature.

[40] Korbel JO, Campbell PJ (2013). Criteria for inference of chromothripsis in cancer genomes. Cell.

[41] Gu S (2016). Mechanisms for complex chromosomal insertions. PLoS Genet..

[42] Ly P, Cleveland DW (2017). Rebuilding chromosomes after catastrophe: emerging mechanisms of chromothripsis. Trends Cell Biol..

[43] Jovtchev G, Stergios M, Schubert I (2002). A comparison of N-methyl-N-nitrosourea-induced chromatid aberrations and micronuclei in barley meristems using FISH techniques. Mutat. Res..

[44] Miller SA, Dykes DD, Polesky HF (1988). A simple salting out procedure for extracting DNA from human nucleated cells. Nucleic Acids Res..

[45] Sánchez-Martín J (2016). Rapid gene isolation in barley and wheat by mutant chromosome sequencing. Genome Biol..

[46] Tarasov A, Vilella AJ, Cuppen E, Nijman IJ, Prins P (2015). Sambamba: fast processing of NGS alignment formats. Bioinformatics.

[47] Simon, A., Pierre, L., Brian, H., & Phil, E. Babraham Bioinformatics—FastQC A Quality Control tool for High Throughput Sequence Data. https://www.bioinformatics.babraham.ac.uk/projects/fastqc/ (2011).

[48] Bolger AM, Lohse M, Usadel B (2014). Trimmomatic: a flexible trimmer for Illumina sequence data. Bioinformatics.

[49] Li H (2009). The sequence alignment/Map format and SAMtools. Bioinformatics.

[50] Quinlan AR, Hall IM (2010). BEDTools: a flexible suite of utilities for comparing genomic features. Bioinformatics.

[51] Chikhi R, Rizk G (2013). Space-efficient and exact de Bruijn graph representation based on a Bloom filter. Algorithms Mol. Biol..

[52] Chikhi R, Medvedev P (2014). Informed and automated k-mer size selection for genome assembly. Bioinformatics.

[53] Sramkoski RM (1999). A new human prostate carcinoma cell line, 22Rv1. Vitro Cell. Dev. Biol. Anim..

[54] Ijdo, J. W., Wells, R. A., Baldini, A. & Reeders, S. T. Improved telomere detection using a telomere repeat probe (TTAGGG)n generated by PCR. **19**, 4780 (1991). 10.1093/nar/19.17.4780 PMC328734 1891373

[55] Ohmido N, Fukui K (1997). Visual verification of close disposition between a rice A genome-specific DNA sequence (TrsA) and the telomere sequence. Plant Mol. Biol..

