# Author Correction: Molecular organization of recombinant human-Arabidopsis chromosomes in hybrid cell lines

**DOI:** 10.1038/s41598-023-35329-8

**Published:** 2023-05-25

**Authors:** Yikun Liu, Yeng Mun Liaw, Chee How Teo, Petr Cápal, Naoki Wada, Kiichi Fukui, Jaroslav Doležel, Nobuko Ohmido

**Affiliations:** 1 Graduate School of Human Development and Environment, Kobe University, Kobe, Hyogo 657-8501 Japan; 2 Centre for Research in Biotechnology for Agriculture, Universiti Malaya, 50603 Lembah Pantai, Kuala Lumpur Malaysia; 3 Institute of Experimental Botany of the Czech Academy of Sciences, Centre of the Region Haná for Biotechnological and Agricultural Research, Šlechtitelů 31, 779 00 Olomouc, Czech Republic; 4 Graduate School of Technology, Industrial and Social Sciences, Tokushima University, Tokushima, Tokushima 770-8503 Japan; 5 Graduate School of Pharmaceutical Sciences, Osaka University, Suita, Osaka 565-0871 Japan

Correction to: *Scientific Reports* 10.1038/s41598-021-86130-4, published online 30 March 2021

The original version of this Article contained an error in the Data availability section, where it did not make clear that the human-Arabidopsis cell line used in the study was obtained from the corresponding author of Ref. 15, and under a license, and therefore cannot be shared by the authors of this Article.

As a result,

"All data generated or analyzed during this study are included in this article and supplemental data." 

now reads:

"All data generated or analyzed during this study are included in this article and supplemental data. The human-Arabidopsis cell line used in the study was used under license from the corresponding author of Ref. 15, and cannot be shared by the authors. Readers wishing to use this cell line for research should direct their requests to the authors of Ref. 15."

The original Article has been corrected.

