# Supplementary material for: Molecular organization of recombinant human-Arabidopsis chromosomes in hybrid cell lines
Source: Sci Rep. 2021 Mar 30;11:7160. doi: 10.1038/s41598-021-86130-4 (PMC8009911; doi:10.1038/s41598-021-86130-4)
Supplement: Supplementary file 1 — Supplementary Information [file 41598_2021_86130_MOESM1_ESM.pdf]

Supplementary information

Molecular organization of recombinant human-Arabidopsis chromosomes in hybrid cell lines

Yikun Liu[1+], Yeng Mun Liaw[1+], Chee How Teo [2], Petr Cápal [3], Naoki Wada [4], Kiichi Fukui [5], Jaroslav Doležel [3], Nobuko Ohmido [1*]

1) Graduate School of Human Development and Environment, Kobe University, Kobe 657-8501, Hyogo, Japan
2) Centre for Research in Biotechnology for Agriculture, University of Malaya, Lembah Pantai, 50603 Kuala Lumpur, Malaysia
3) Institute of Experimental Botany of the Czech Academy of Sciences, Centre of the Region Hana for Biotechnological and Agricultural Research, Šlechtitelů 31, 779 00 Olomouc, Czech Republic
4) Graduate School of Technology, Industrial and Social Sciences, Tokushima University, Tokushima 770-8503, Tokushima, Japan
5) Graduate School of Pharmaceutical Science, Osaka University, Suita 565-0871, Osaka, Japan

Table S1 Fusion breakpoint region (marked by *) between Arabidopsis chromosomes 5 and 2 identified using PCR (262 bp)

chr5-2 fusion junction
AGATGTGGAGAGTGATTTCATCGAAGTATAGTCTTATAATAAGACTTTCCTTGTTTG<br>CACTAATTTTTTTTCTTTCTGGCATATAAAAAATTATGGAATGAAACATATTAAAAAT<br>ACGTCAGAACGCCGAGTTTTCCAATGTTAATAAGTAGAGAATGTGTGTAAATATGT<br>TTCTTGGAAAAGAAGTAAGATGATGGTT*GTTTGGTATGTTTTTCTTCTCTGTATTCC<br>ATCAGCTATTATTTCTCCTCTCTCCAATAATATT

Table S2 Satellite DNA composition in genomes masked against the Arabidopsis repeat library

Genome	Number of elements	Total length (bp)	Proportion of the genome (%)
Hybrid cell line, 60 days	15432	4879562	0.17
Hybrid cell line, 300 days	12761	3740017	0.13
Arabidopsis (TAIR10)	3146	976448	0.82
Human (GRCh38)	1256	1485509	0.05

Table S3 Names and locations of the Arabidopsis-specific probes used for FISH

Probe	Chromosome position	Chromosomal location (bp)	Length (bp)
Ch2-1	Ch2	10050013-10050444	432
Ch2-2	Ch2	10052311-10052774	464
Ch2-3	Ch2	10053004-10053584	581
Ch2-4	Ch2	10054381-10054793	413
Ch2-5	Ch2	10057397-10057806	410
Ch3-1	Ch3	2328346-2328816	471
Ch3-2	Ch3	2329574-2330055	482
Ch3-3	Ch3	2332128-2332618	491
Ch3-4	Ch3	2337162-2337569	408
Ch3-5	Ch3	2338472-2338908	427
Ch5-1	Ch5	10214988-10215445	458
Ch5-2	Ch5	10217345-10217791	447
Ch5-3	Ch5	10217872-10218357	486
Ch5-4	Ch5	10219147-10219548	402
Ch5-5	Ch5	10220357-10220843	487

Table S4 PCR primers used to prepare FISH probes to detect fusion breakpoints in the neo-chromosome

**Primer**	**Sequence (5′→3′)**	**Length**
Ch2-1	Forward: GGTTGGGAGATCACTTCAGG<br>Reverse: CAAGTCAAGGGGAAACCAAA	432 bp
Ch2-2	Forward: TAATGGAGCCCAGGTAGTGC<br>Reverse: CGTGGACGAGCATATTGGTA	464 bp
Ch2-3	Forward: TGCCTTGAAACAGCATCAAC<br>Reverse: CGAACAATTTCGTGCAACAC	581 bp
Ch2-4	Forward: ACCAAAAGCACCACTTGCTC<br>Reverse: CAAGCTTGACGAACCTTTCC	413 bp
Ch2-5	Forward: TCACCACAACCTTACCACGA<br>Reverse: TGTCGATGTTGAGACCAGGA	410 bp
Ch3-1	Forward: TGCTTGTTGGAGATCTGTGC<br>Reverse: GGTTGGCAGAAGGATAGCTG	471 bp
Ch3-2	Forward: CTCTTCGCCGGAATAACAAA<br>Reverse: GAAATTTCCAGGTGGCTCAA	482 bp
Ch3-3	Forward: CATTATTCGCGGGAAAAGAA<br>Reverse: ACCTGATTTGACCGTTTTGC	491 bp
Ch3-4	Forward: CTCAACAGTTGTTCGCTCCA<br>Reverse: AGTTGGCAGTGGGAAAAATG	408 bp
Ch3-5	Forward: AACCACCAAGCGTAAACCAC<br>Reverse: GGTGGAACAAGCAACGAGAT	427 bp

Ch5-1	Forward: TATGCCCTGTCCAAGACTCC<br>Reverse: TGGACTGGTCGTCCCTATTT	458 bp
Ch5-2	Forward: CATCGGCAGAACATTGATTG<br>Reverse: CCTCTCTACTCGCCGACAAC	447 bp
Ch5-3	Forward: TGGGGTTTTTAGGTTGACCA<br>Reverse: GCTTTCCGCTTTTCTCATTG	486 bp
Ch5-4	Forward: AGCGGGCATTCATCATTAAC<br>Reverse: TCAGCAACAAAAACCCATCA	402 bp
Ch5-5	Forward: GAGAGGTGGCAAAGCAAAAG<br>Reverse: ACTGCCCTCGGACCTATTTT	487 bp
Arabidopsis Telomere	Forward: TTTAGGGTTTAGGGTTTAGGG<br>Reverse: CCCTAAACCCTAAACCCTAAA	7n bp
AtCen	Forward: GATCAAGTCATATTCGACTC<br>Reverse: GTTGTCATGTGTATGATTGA	180 bp
Human Telomere	Forward: TTAGGGTTAGGGTTAGGGTTAGGGTTAGGG<br>Reverse: CCCTAACCCTAACCCTAACCCTAACCCTAA	6n bp
Chr 5-2 fusion junction	Forward: TGACTACTTAATTAGATGTGGAGAGTG<br>Reverse: GGACACAACTATAAGCGCAAG	166 bp

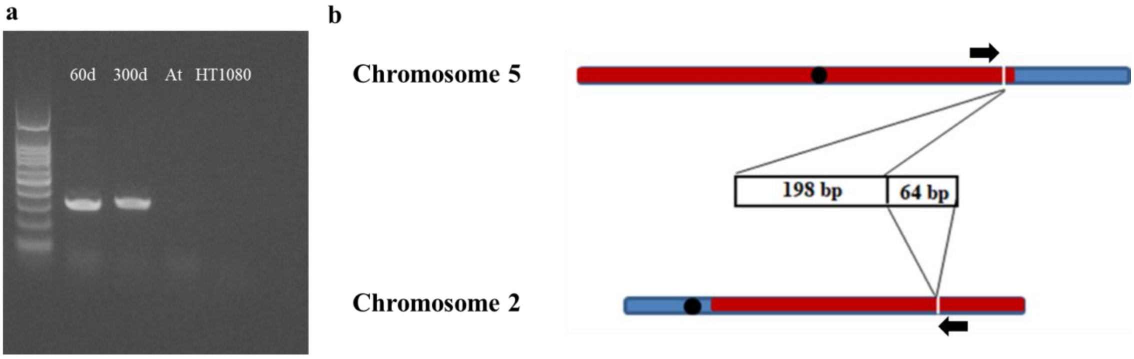

Figure S1 Identification of a fusion breakpoint between fragments of Arabidopsis chromosomes 2 and 5 in the human-Arabidopsis hybrid cell line. (**a**) PCR results of the fusion junction in 60- and 300-day-old (60d and 300d respectively) hybrid cells with Arabidopsis and human cell line HT1080 as the negative control. (**b**) Schematic diagram depicting the fusion breakpoint. Black arrow indicates orientation of primers.

Note; The original full-length gels and blots image is following.

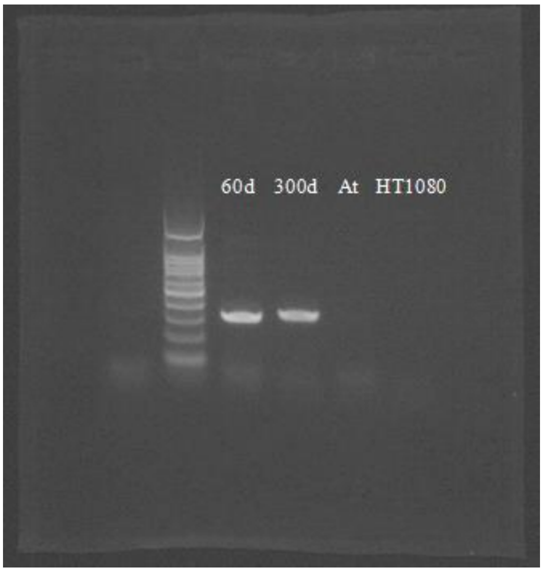

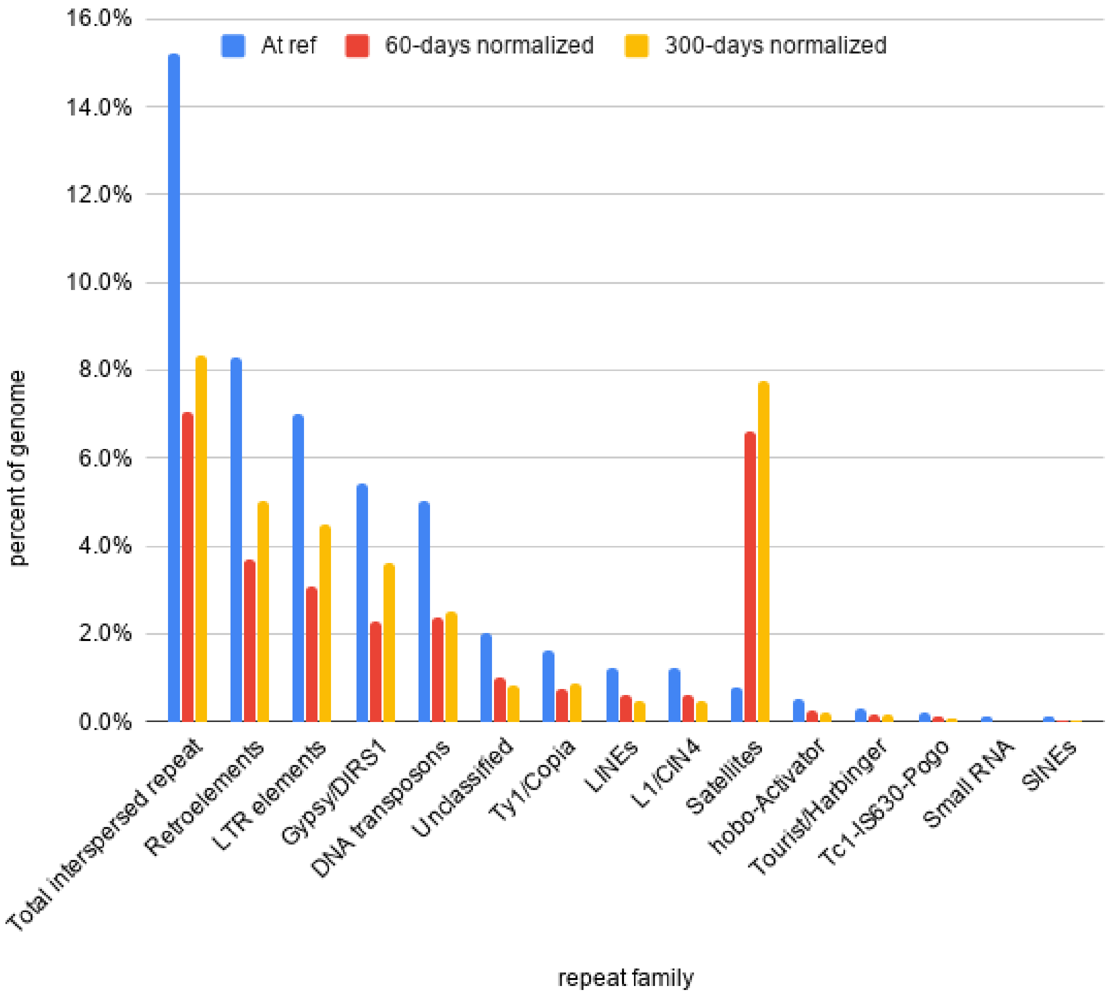

Figure S2 Repeat abundance in the genomes of wild-type Arabidopsis (At ref), and 60- and 300-day-old hybrid cells (normalized against human background) relative to the percentage of Arabidopsis genome present.

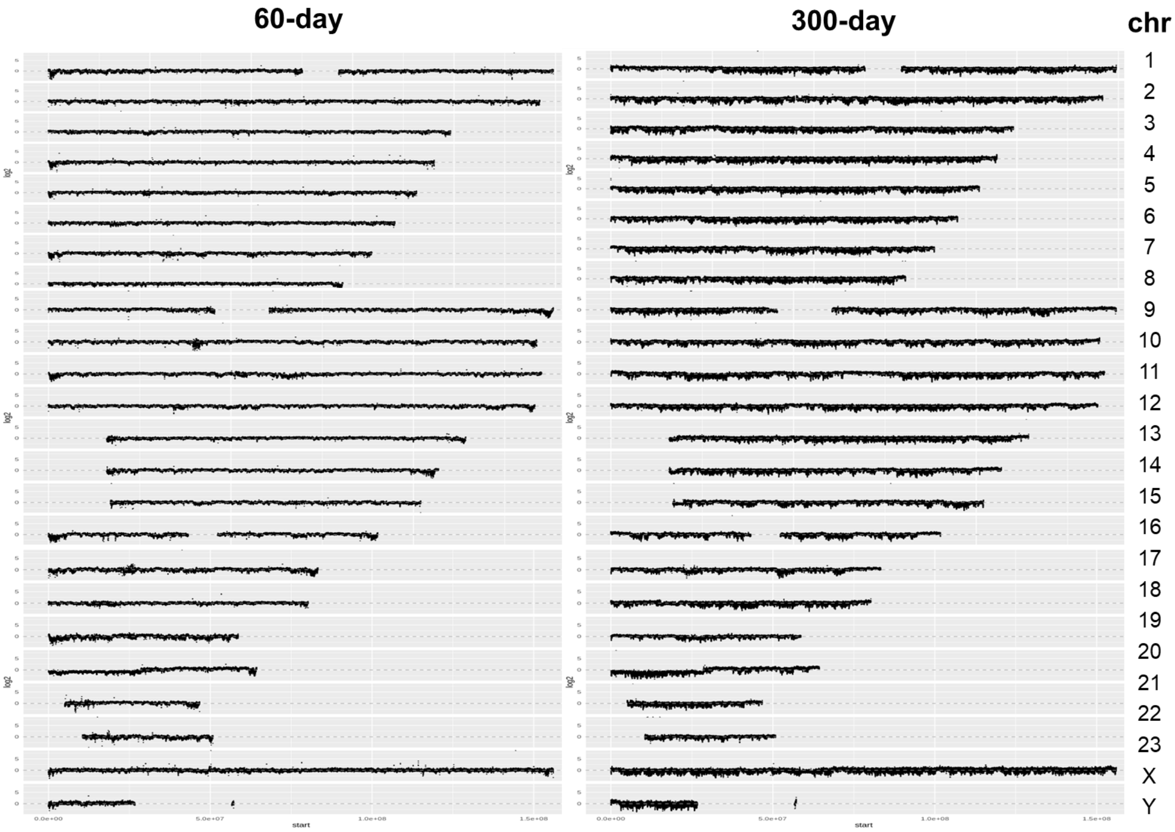

Figure S3 Copy number variation of human chromosomes in the 60- and 300-day-old hybrid cell lines. Log2 ratio of zero (gray dashed line) indicates diploid level. X-axis: chromosome position; Y-axis: log2 ratio relative to HT1080.

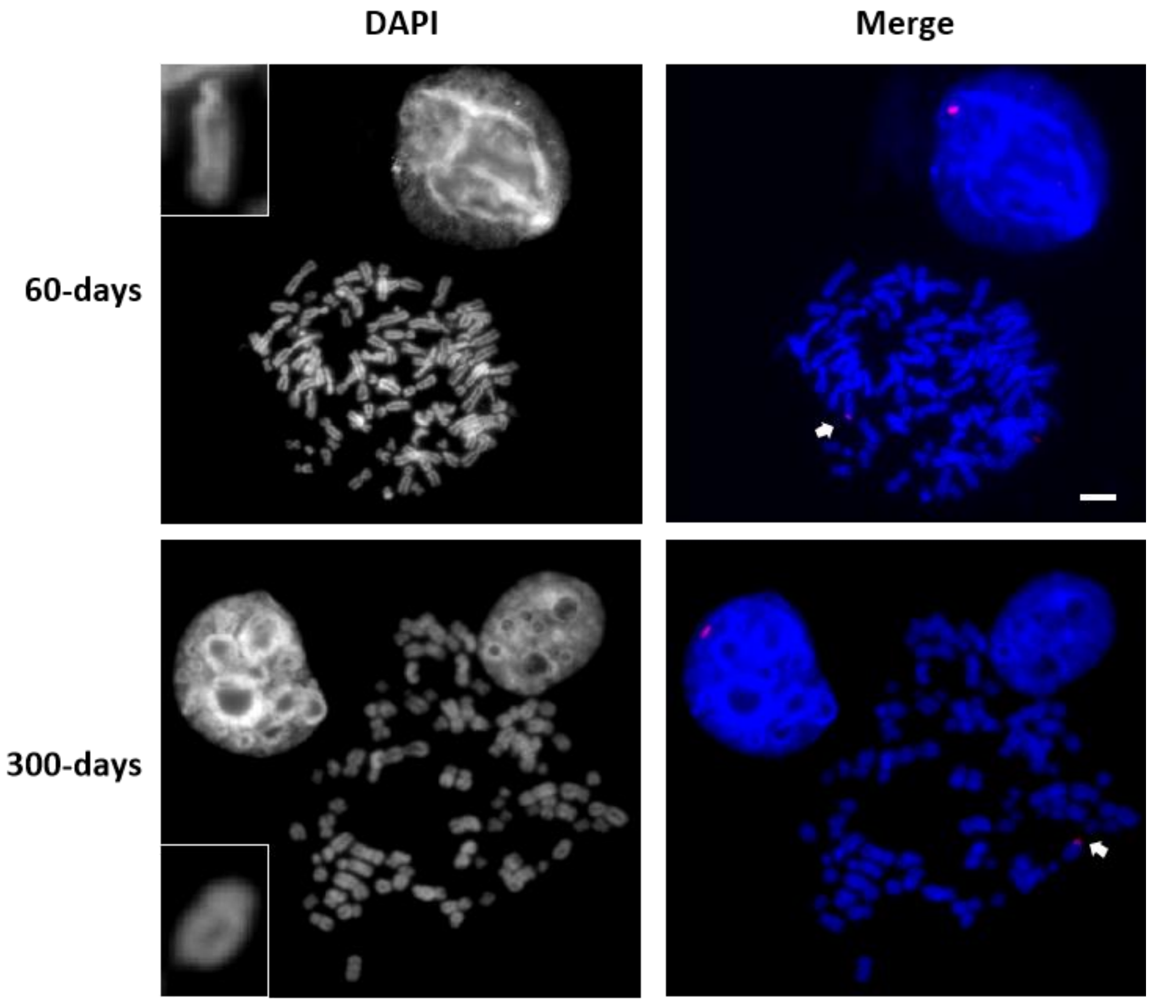

Figure S4 FISH images with Arabidopsis centromeric probe (red) of 60- and 300-day-old hybrid cell line. Scale bars, 5 μm.

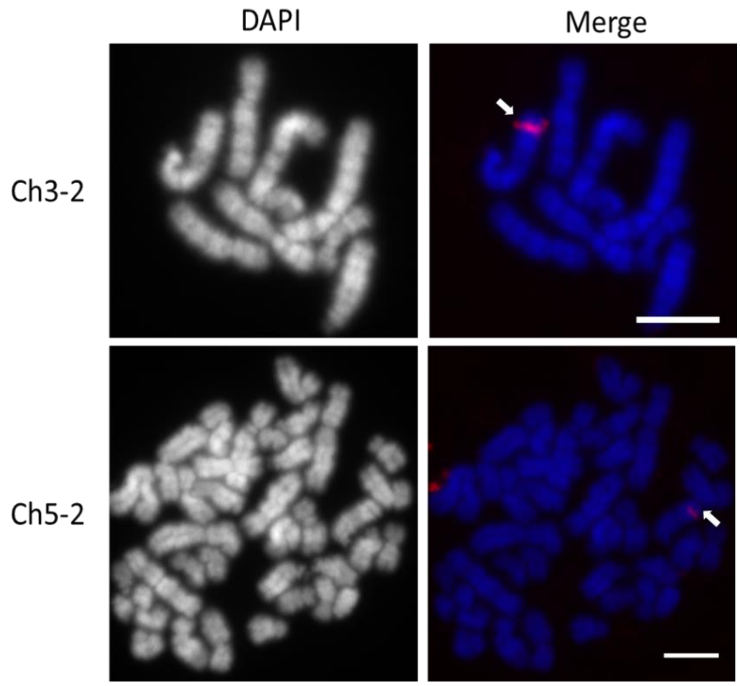

Figure S5 FISH images with probes Ch3-2 (red) and Ch5-2 (red) on metaphase chromosomes of the 300-day-old hybrid cell line. Scale bars, 5 μm.

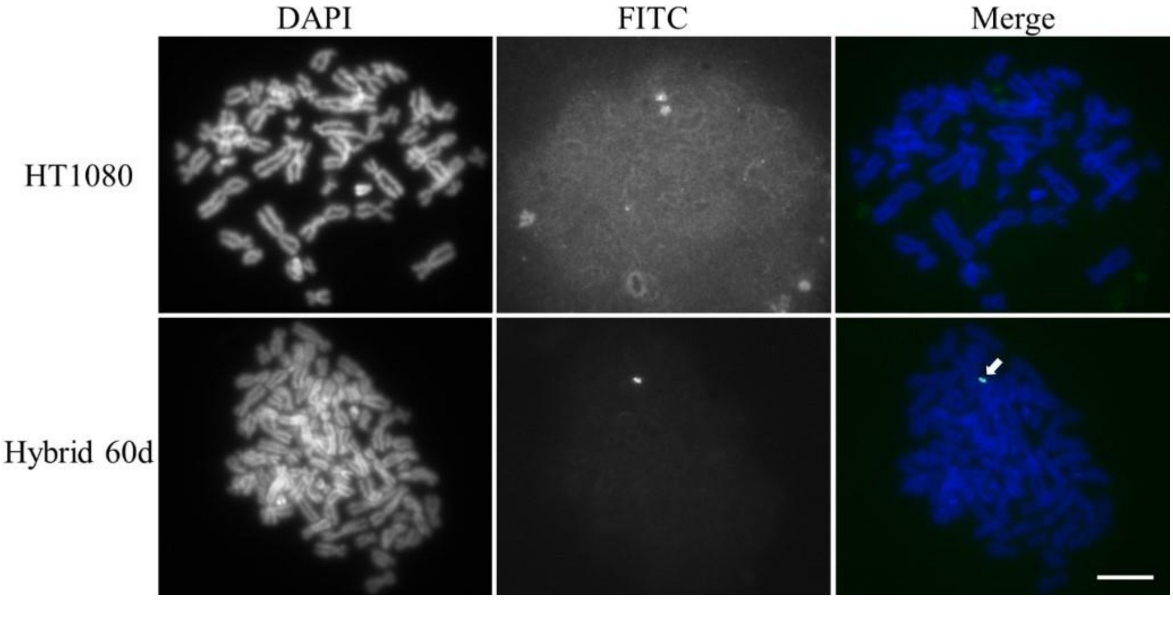

Figure S6 FISH with a probe for Ch2-2 (green) in mitotic metaphase plates of the HT1080 cell line and the 60-day-old hybrid cell line. Scale bar, 5 μm.
